# Diabetes and Heart Failure: Multi-Omics Approaches

**DOI:** 10.3389/fphys.2021.705424

**Published:** 2021-08-06

**Authors:** Akram Tayanloo-Beik, Peyvand Parhizkar Roudsari, Mostafa Rezaei-Tavirani, Mahmood Biglar, Ozra Tabatabaei-Malazy, Babak Arjmand, Bagher Larijani

**Affiliations:** ^1^Cell Therapy and Regenerative Medicine Research Center, Endocrinology and Metabolism Molecular-Cellular Sciences Institute, Tehran University of Medical Sciences, Tehran, Iran; ^2^Metabolomics and Genomics Research Center, Endocrinology and Metabolism Molecular-Cellular Sciences Institute, Tehran University of Medical Sciences, Tehran, Iran; ^3^Proteomics Research Center, Shahid Beheshti University of Medical Sciences, Tehran, Iran; ^4^Endocrinology and Metabolism Research Center, Endocrinology and Metabolism Clinical Sciences Institute, Tehran University of Medical Sciences, Tehran, Iran; ^5^Non-Communicable Diseases Research Center, Endocrinology and Metabolism Population Sciences Institute, Tehran University of Medical Sciences, Tehran, Iran

**Keywords:** diabetes mellitus type 2, heart failure, diabetic cardiomyopathies, metabolomics, oxidative stress

## Abstract

Diabetes and heart failure, as important global issues, cause substantial expenses to countries and medical systems because of the morbidity and mortality rates. Most people with diabetes suffer from type 2 diabetes, which has an amplifying effect on the prevalence and severity of many health problems such as stroke, neuropathy, retinopathy, kidney injuries, and cardiovascular disease. Type 2 diabetes is one of the cornerstones of heart failure, another health epidemic, with 44% prevalence. Therefore, finding and targeting specific molecular and cellular pathways involved in the pathophysiology of each disease, either in diagnosis or treatment, will be beneficial. For diabetic cardiomyopathy, there are several mechanisms through which clinical heart failure is developed; oxidative stress with mediation of reactive oxygen species (ROS), reduced myocardial perfusion due to endothelial dysfunction, autonomic dysfunction, and metabolic changes, such as impaired glucose levels caused by insulin resistance, are the four main mechanisms. In the field of oxidative stress, advanced glycation end products (AGEs), protein kinase C (PKC), and nuclear factor kappa-light-chain-enhancer of activated B cells (NF-κB) are the key mediators that new omics-driven methods can target. Besides, diabetes can affect myocardial function by impairing calcium (Ca) homeostasis, the mechanism in which reduced protein phosphatase 1 (PP1), sarcoplasmic/endoplasmic reticulum Ca2+ ATPase 2a (SERCA2a), and phosphorylated SERCA2a expressions are the main effectors. This article reviewed the recent omics-driven discoveries in the diagnosis and treatment of type 2 diabetes and heart failure with focus on the common molecular mechanisms.

## Introduction

Diabetes mellitus is defined as a major metabolic disorder that is associated with considerable and long-term microvascular and macrovascular complications (Adeghate and Singh, 2014). Herein, heart failure (HF) has been identified in patients with diabetes since 1876 (Lee et al., 2019). Indeed, cardiovascular (CV) disease is a primary cause of disability and death due to diabetes (Boudina and Abel, 2010). “Diabetic cardiomyopathy” (DC) can be manifested by diastolic dysfunction, cardiomyocyte hypertrophy, and apoptosis along with myocardial fibrosis (Huynh et al., 2014). Moreover, DC contributes to the higher incidence of HF in patients with diabetes (Aneja et al., 2008). Among different underlying mechanisms that are common between type 2 diabetes mellitus (T2DM) and HF, oxidative stress is a key contributor. Coenzyme Q (CoQ)10 supplementation and gene therapy, as well as targeting cardiac phosphoinositide-3-kinase (PI3K) (p110α) signaling, protein kinase-C (PKC) signaling, and dysregulated microRNAs (miRNAs), are newer promising therapeutic approaches (Huynh et al., 2014). On the other hand, the development of high-throughput techniques utilizing multi-omics data have provided a holistic study on complex biological processes, especially in disease subtyping and providing biomarkers (Subramanian et al., 2020). Although there are some challenges in utilizing multi-omics technologies, they are currently being used to uncover underlying biological pathways of disorder and molecular basis of complex phenotypes at different dimensions (Chakraborty et al., 2018), which are applied in different disorders such as cancer (Chaudhary et al., 2018; Yoo et al., 2018), CV diseases (V et al., 2015; Leon-Mimila et al., 2019), and diabetes. In this review, we first stated different aspects of T2DM as a chronic lifelong disease through its clinical features, consequences, epidemiology, and prognosis, especially concomitant with HF and CV events. Then, we explained the coexistence of T2DM and HF, focusing on their common underlying pathways. Lastly, we introduced omics studies as promising therapeutic technologies particularly targeting common mechanisms of these two major disorders.

## T2DM: A Chronic Lifelong Disease

Diabetes mellitus is an old human disorder that was first mentioned about 3,000 years ago. T2DM, the most common type of DM, was first reported in 1988 as part of metabolic syndrome (Olokoba et al., 2012). It is an important cause of mortality because of its associated CV complications and several other pathogenetic disturbances (Blaslov et al., 2018). Age, race, and ethnicity as well as physical activity, diet, and smoking can be linked to T2DM etiologies (Sami et al., 2017). However, the underlying direct pathological mechanism of T2DM is complex and has many different elements (Leahy, 2005). More knowledge of the pathophysiological mechanisms of T2DM can lead to better prediction, earlier diagnosis, and improved therapeutic approaches (Ma et al., 2018). Some other features of T2DM regarding its clinical characteristics, consequences, prognosis, and epidemiological aspects are explained in the next subsections.

### Clinical Features and Consequences

First, there are some metabolic, genetic, and environmental risk factors that predispose individuals to T2DM. Overweight, CV events, hypertension, and dyslipidemia are some of the important risk factors for T2DM (Fletcher et al., 2002; Vijan, 2010). T2DM diagnosis can be performed by the measurement of venous plasma glucose and hemoglobin A1c (HbA1c), which are standardized and quality-assured laboratory approaches (Kerner and Brückel, 2014). As stated, most patients with diabetes have the T2DM form of the disease, which can have an asymptomatic and latent period of sub-clinical stages (DeFronzo et al., 2015). However, T2DM at very high stages of hyperglycemia can be accompanied by some symptoms such as polyuria, polydipsia, and polyphagia, which are classic symptoms of the disease (Ramachandran, 2014). Diabetes has a mentionable association with microvascular and macrovascular complications that can lead to organ damage (Cade, 2008; Chatterjee et al., 2017). It should be stated that cardiovascular autonomic neuropathy (CAN) has a major role in diabetic autonomic neuropathy complications (Vinik et al., 2003). Also, endothelium dysfunction due to DM can lead to other CV events, which predominantly affect coronary, peripheral, and carotid arteries (Stolar and Chilton, 2003). It is mentioned that intensive glycemic control has lesser effect on macrovascular complications compared with microvascular sequelae (Chatterjee et al., 2017).

### Epidemiology and Prognosis

Diabetes mellitus as an epidemic of the century along with its accompanying complications has a major global health impact on economies. The number of patients with DM has quadrupled globally over the last three decades, making it a major concern worldwide. This estimation is predicted to rise even to 642 million by 2040 (Zheng et al., 2018). The significantly high burden of the disease can be seen in some specific regions of the world (island states of the Pacific, Western Europe) (Khan et al., 2020). CV complications are mentioned to be the major reasons for morbidity and mortality due to DM (Zheng et al., 2018). It has been estimated that 1% higher glycosylation of Hb is associated with about 8% higher risk for HF (Cas et al., 2015). HF can be mentioned as an independent risk factor for developing T2DM (Bell and Goncalves, 2019), and patients with diabetes are also at higher risk for HF development following myocardial infarction (Stone et al., 1989). Thus, the increased prevalence of HF can be seen in patients with diabetes along with worse prognosis (Lehrke and Marx, 2017; Norhammar et al., 2017; Zareini et al., 2018; Bell and Goncalves, 2019). Diabetes can also lead to worse outcomes of acute coronary syndromes in the early and late stages of the disease (Beckman et al., 2002). Moreover, other mentioned macrovascular and microvascular complications of DM can also result in death and reduce the quality of life through blindness, kidney failure, peripheral neuropathy, and several other consequences (Bailes, 2002; Cole and Florez, 2020). T2DM prevalence and incidence are still increasing, to which serious attention should be paid because of its severe consequences (Khan et al., 2020).

## Coexistence of T2DM and HF

**FIGURE 1:**
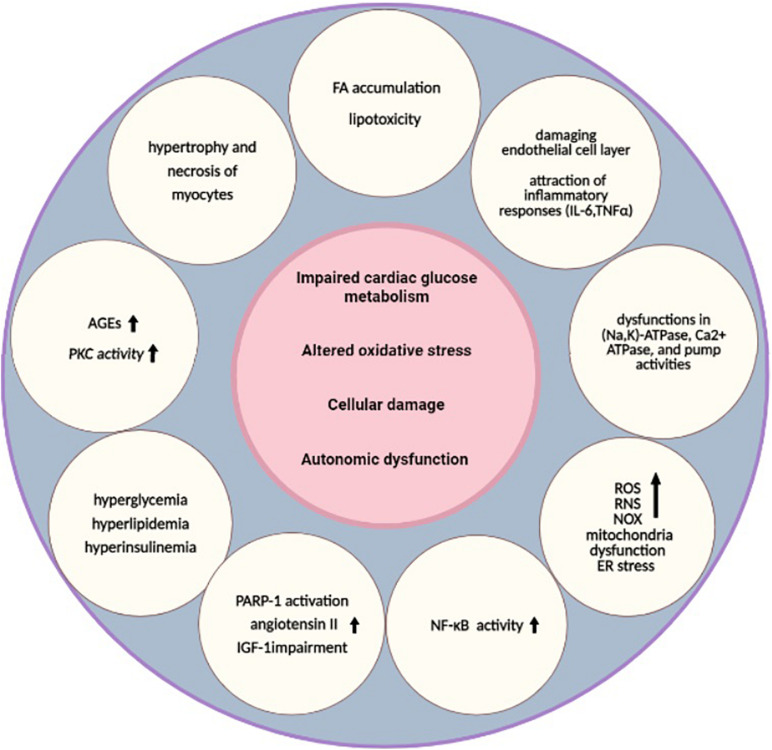
Underlying pathways of diabetic cardiomyopathy. Impaired cardiac glucose metabolism (Mortuza and Chakrabarti, 2014), altered oxidative stress (Khullar et al., 2010), cellular damage (Cai and Kang, 2003), and autonomic dysfunction are the main pathological mechanisms that are common between T2DM and HF (Rodriguez-Saldana, 2019). Several other alterations, such as hyperglycemia, hyperlipidemia, hyperinsulinemia (Borghetti et al., 2018), higher AGEs, increased PKC activity (Brahma et al., 2017; Kaludercic and Di Lisa, 2020), FA accumulation, lipotoxicity (Maisch et al., 2011), damaged endothelial cell layer, attraction of inflammatory responses (IL-6, TNFα) (Lorenzo et al., 2011), higher NF-κB activity (Faria and Persaud, 2017), dysfunctions in (Na,K)-ATPase, Ca2+ ATPase, and pump activities, depressed CK activities (Cai and Kang, 2001), increased levels of ROS, RNS, NOX (Liu et al., 2014), mitochondria dysfunction (Varma et al., 2018), ER stress, hypertrophy and necrosis of myocytes (Jia et al., 2018), PARP-1 activation, increased angiotensin II, and IGF-1 impairment, are also related to these pathways (Bugger and Abel, 2014). These alterations can lead to diabetic cardiomyopathy as a result. HF, heart failure; AGEs, advanced glycation end products; ATP, adenosine triphosphate; CK, creatine kinase; ER, endoplasmic reticulum; FA, fatty acids; IGF-1, insulin-like growth factor 1; IL, interleukin; NF-κB, nuclear factor kappa-light-chain-enhancer of activated B cells; NOX, nicotinamide adenine dinucleotide phosphate-oxidases; PARP-1, poly [ADP-ribose] polymerase 1; PKC, protein kinase C; RNS, reactive nitrogen species; ROS, reactive oxygen species; T2DM, type 2 DM; TNF, tumor necrosis factor.

Heart failure is a life-threatening clinical syndrome in which the heart is unable to provide blood flow sufficiently and could not meet metabolic requirements (Kemp and Conte, 2012). Pathogenesis mechanisms of HF in diabetes can be related to hypertension, cardiotoxic tetrad related to coronary artery disease, and extracellular fluid volume expansion (Gilbert and Krum, 2015). “DC” is the term that is used for the presence of myocardial dysfunction in the absence of CV associated risk factors (Lehrke and Marx, 2017). The metabolism, function, and structure of the cardiac system may be affected by the underlying pathways of both diabetes and HF (Wallner et al., 2018). In the early stages of DC, structure and morphology changes are not considerable, but in the late stages, both systolic and diastolic function may be affected with greater increases in size and wall thickness of the left ventricular mass (Jia et al., 2016). In the next subsections, diabetes and HF as two major diseases are brought together considering their common mechanisms and underlying molecular pathways (Figure 1).

### Impaired Cardiac Glucose Metabolism

Hyperglycemia is mentioned to have important roles in triggering molecular and cellular pathways of diabetes (Mortuza and Chakrabarti, 2014; Borghetti et al., 2018). According to many reports, diabetes can cause abnormalities in the heart tissue directly without the necessity of vascular defects. In a diabetic state, impairments in glucose uptake and glycolysis and abnormalities in pyruvate oxidation, along with impairment of insulin function, can promote lipolysis and fatty acid (FA) release from adipose tissues. Thus, these events could lead to the development of cardiomyopathy because of the adaption of cardiac muscle to exclusive utilization of FA for ATP generation (An and Rodrigues, 2006). Indeed, accumulation of FA and lipotoxicity can affect heart function due to altered lipid signaling (Maisch et al., 2011). Taken together, higher FA metabolism, decreased amount of protective glucose metabolism, and insulin resistance along with neurohumoral activation can lead to some perturbations in myocardial energetic functions (Salabei et al., 2016). Also, activation of other pathways, such as increased polyol flux, increased advanced glycation end products (AGEs), and higher activity of PKC, in addition to higher mitochondrial dysfunction and oxidative stress can lead to the development of DC as a result of hyperglycemia. The induction of *O*-linked *N*-acetylglucosamine (*O*-GlcNAc) modification (through increased hexosamine pathway) may result in altered Ca2+ sensitivity and cycling and, thus, impaired cardiac protein contractility (Brahma et al., 2017; Kaludercic and Di Lisa, 2020). Also, several studies have also suggested the effects of hyperglycemia in inducing apoptotic cell death (Cai et al., 2002). All events of glucose metabolism are related to higher oxidative stress, which contains the basis metabolic impairments of DC (Mandavia et al., 2013), which is explained in the following section.

### Altered Oxidative Stress

Oxidative stress is stated as one of the most important causes for DC pathophysiology, which contributes to both onset and complications of diabetes (Khullar et al., 2010; Liu et al., 2014; Ding et al., 2019). Increased production of ROS and reactive nitrogen species (RNS) in mitochondria can be derived from hyperglycemia in both main types of diabetes. Also, lack of insulin-mediated glucose metabolism can result in increased FA concentrations that can cause higher production of ROS from nicotinamide-adenine-dinucleotide-phosphate (NADPH)-oxidases (NOX). The activation of NOX results in higher production of superoxide, which, in combination with nitric oxide (NO), can produce damaging peroxynitrite (Liu et al., 2014). Indeed, transport of FA through CD36 can activate PKC-2β, leading to NOX2 activation and promoting recruitment of NOX2 catalytic subunits, and superoxide production, inducing a positive feedback loop of ROS production (Hansen et al., 2018). Cardiac dysfunction can be also related to mitochondria uncoupling along with mitochondrial-electron transport chain leakage. Also, the activation of PKC, xanthine oxidase, and lipoxygenases has a role in ROS production in a diabetic heart (Varma et al., 2018). PKC-dependent activation of the reduced form of NADPH has roles in inducing cellular ROS (Lee et al., 2004). Indeed, promoting recruitment of NOX2 catalytic subunits and superoxide production have positive effects on ROS production (Hansen et al., 2018). Activation of GTP-binding protein Rac-1 takes part in this induced NAD(P)H oxidase activation (Inoguchi et al., 2003). Xanthine oxidase, a cytoplasmic enzyme, catalyzes the oxidation of hypoxanthine and xanthine (its substrates) to uric acid utilizing O^2^ (as an electron acceptor), leading to O^2^− and H_2_O_2_ production. This may present as an important source of ROS production in cardiomyocytes (Kayama et al., 2015). Lipoxygenases contain lipid-peroxidizing enzymes that form hydroperoxy derivatives by oxidizing free and esterified polyunsaturated FAs (Kühn and O’Donnell, 2006). Herein, oxidative damage of proteins, lipids, and DNA resulted from an imbalance in ROS production (Varma et al., 2018). Other abnormalities associated with ROS production include some dysfunctions in (Na,K)-ATPase, Ca2+ ATPase, and pump activities in addition to depressed creatine kinase (CK) activities of the heart. On the other hand, in diabetic rats, CoQ, which has a potential antioxidant activity, is shown to be decreased (particularly CoQ9 and CoQ10) in cardiac mitochondria (Cai and Kang, 2001). Additionally, toxic molecules in the oxidative stress state affect Ca2+ handling and subcellular remodeling. Impaired left ventricular function can be the result of decreased Ca2+ sensitivity, reduced sarco(endo)plasmic reticulum Ca2+ -ATPase (SERCA2a) activity, and shifting myosin heavy chain. All of these could as a result lead to DC (Trost et al., 2002; Suarez et al., 2008; Goyal and Mehta, 2013; Zarain-Herzberg et al., 2014). The SERCA pump plays a part in muscle relaxation by means of lowering Ca2+ and also in restoration of sarcoplasmic reticulum (SR) Ca2+ load for muscle contraction. SERCA2a pump function is affected by inhibitory peptide phospholamban. Increasing the levels of this inhibitory function along with higher activity of protein phosphatase 1 (PP1) may result in inactivation/dephosphorylation of protein kinase A (PKA) targets related to SR Ca2+ uptake dysfunctions (Lipskaia et al., 2010; Zarain-Herzberg et al., 2014). On the other hand, increased ROS along with higher glucose levels and lipid changes leads to damaged endothelial layer and attraction of inflammatory responses. Cytokines such as interleukins (IL)-6 and tumor necrosis factor (TNF)α, adhesion molecules, and angiotensin-II are released from the endothelial cell layer, which causes migration of more leukocytes to subendothelial layers of inflammation. It could result in fibrosis and atherosclerotic plaque in which nuclear-factor kappa-light-chain-enhancer of activated B cells (NF-κB) has an important regulatory role (Lorenzo et al., 2011). Thus, NF-K b can also be activated following oxidative stress, which causes cardiac fibrosis and hypertrophy. It can also lead to inflammation and excessive oxidative stress related to DNA and membrane injury (Faria and Persaud, 2017).

### Cellular Damage

Myocardial cell death has important effects on the pathophysiology of different cardiomyopathies, such as endothelial dysfunction, myocardial infarction, and DC. In this regard, ROS and RNS have important roles in inducing different apoptotic signaling pathways (Cai and Kang, 2003). Also, biochemical changes such as hyperglycemia in DC can lead to thickening of capillary-basement membrane as well as hypertrophy and necrosis of myocytes (Farhangkhoee et al., 2006). On the other hand, endoplasmic reticulum (ER) stress can result in unfolded protein reaction and its proteasomal degradation. ER stress could promote apoptosis and cellular damage and lower the function of sarcoplasmic reticulum-calcium pump, which takes part in Ca2+ sequestration (Yang et al., 2015; Jia et al., 2016). Indeed, ER stress in combination with oxidative stress and impaired calcium handling may lead to apoptosis, autophagy, and cellular necrosis (Jia et al., 2018). Moreover, normal autophagy can be affected by some impairments of autophagosome and lysosome fusion, which influences both the diastolic and systolic functions of the diabetic heart (Xie et al., 2011). The renin-angiotensin-aldosterone system (RAAS) also has notable roles in the progression of DC through higher oxidative damage along with cellular necrosis and apoptosis of diabetic heart (Murarka and Movahed, 2010). On the other hand, apoptotic cell death can be explained by higher inflammatory cytokines as well as Fas receptor-dependent apoptosis pathways. Excessive activation of poly [ADP-ribose] polymerase 1 (PARP-1) and impairment of insulin-like growth factor 1 (IGF-1), in addition to higher levels of angiotensin II, can also induce cellular necrosis pathways (Bugger and Abel, 2014). It should be noted that PARP-1 is involved in different physiological pathways such as cell death and DNA repair. Impaired DNA can activate PARP-1, which results in the cleaving of NAD+ into nicotinamide and ADP-ribose. Overactivation of PARP-1 can lead to irreversible cytotoxicity, cellular damage, and even death as a result of higher NAD+ and ATP depletion (Qin et al., 2016). On the other hand, caspase-independent cell death can be triggered by this enzyme, termed parthanatos, which is mentioned to be distinct from apoptosis/necrosis (or autophagy). This PARP-1-mediated cell death can be induced by apoptosis-inducing factor (AIF) nuclear translocation (Bugger and Abel, 2014).

### Autonomic Dysfunction

Cardiovascular autonomic dysfunction can be defined as impaired autonomic control of the CV system in the diabetic state when other causes are excluded. Damages to nerve fibers can cause this abnormality of the CV system. There are several interactions of pathogenic pathways that have roles in CAN in which hyperglycemia is introduced as a major and initial cause. Oxidative/nitrosative stress, ER stress, impaired mitochondrial function and membrane permeability, inflammation, and calcium imbalance can be involved in this CAN pathway (Rodriguez-Saldana, 2019). Taken together, autonomic diabetic neuropathy as an important complication of DM can affect different organs (especially the CV system). As mentioned earlier, CAN can cause some clinical/functional manifestations that can be brought about by some alterations in vascular dynamics and uncontrolled heart rate (Flotats and Carrió, 2010). Hypertension, exercise intolerance, QT interval prolongation, and higher arterial stiffness are also associated with CAN. The peripheral vascular function can be also affected by this autonomic process (Rodriguez-Saldana, 2019). This autonomic dysfunction can predict CV risk and contributes to poor prognosis, higher mortality rates, and sudden death (Flotats and Carrió, 2010; Stables et al., 2013; Vinik et al., 2013). It has been found that there is an association between the dysfunction degree of the left ventricle and levels of cardiac autonomic dysfunction (Poirier et al., 2003). It has also been found that metabolic factors have important effects on this autonomic process (Valensi et al., 1997). Indeed, CAN could be seen early in the diabetes state, which can be a prognostic factor for microangiopathic complications (Valensi et al., 2003).

## Brief Review of Omics Studies

**FIGURE 2:**
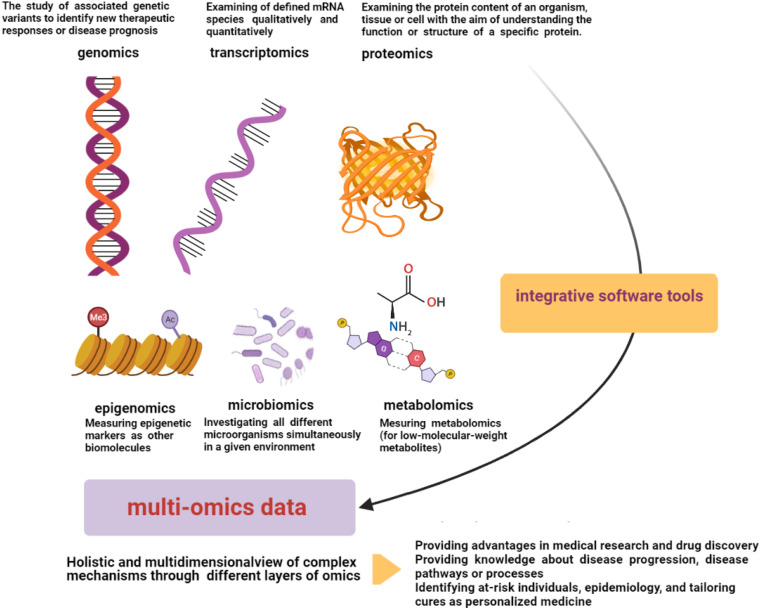
Multi-omics data. Multi-omics technologies through different layers of omics data (genomics, transcriptomics, microbiomics, proteomics, epigenomics, and metabolomics) can provide a multidimensional analysis network to develop knowledge of interactive molecular basis and complex underlying pathophysiology of disorders. Electronic health records, imaging, and other integrative software tools can be helpful in integrative processes (Sun and Hu, 2016; Hasin et al., 2017).

“OMICS” strategies are defined by providing high-throughput interfaces (in global-unbiased ways) to investigate millions of markers that represent similar biochemical identities simultaneously. These technologies can be used to find out the underlying molecular properties that exist behind complex phenotypes (Chakraborty et al., 2018; Conesa and Beck, 2019). There are several data types of omics technologies including genomics, transcriptomics, microbiomics, proteomics, epigenomics (Hasin et al., 2017; Manzoni et al., 2018), and metabolomics (Sun and Hu, 2016; Figure 2). Among the omics technologies, genomics, the most mature field, is the study of associated genetic variants to identify new therapeutic responses or disease prognosis. The human genome has important roles in personalized medicine with the aims of disease treatment and prevention considering genetic susceptibility (Burke and Psaty, 2007; Hasin et al., 2017; Ahmed, 2020). In this regard, the effects of genetic variant knowledge regarding genome-wide-association-studies (GWASs) along with omics findings have been found (Akiyama, 2021). Transcriptomics, another high-throughput technology, is responsible for the simultaneous examination of defined mRNA species qualitatively and quantitatively (Hegde et al., 2003). Proteomics is also another omics-related technology with the ability to examine the protein content of an organism, tissue, or cell with the aim of understanding the function or structure of a specific protein. This technology can be used in various research settings with different capacities to find diagnostic markers, vaccine production, and even interpretation of protein pathways of disorders (Aslam et al., 2017). Herein, large-scale protein characterization in this high-throughput proteomics strategy may benefit from MS-based technique (Bruce et al., 2013; Zhang et al., 2014). Moreover, other related technologies have been found to measure other biomolecules. For instance, epigenomics (for epigenetic markers) or metabolomics (for low-molecular-weight metabolites) can be utilized in these fields (Sun and Hu, 2016). Microbiomics is another developed omics-related technology that investigates all different microorganisms simultaneously in a given environment (Wong et al., 2015; Hasin et al., 2017). Thus, omics technologies have different layers for a comprehensive study on a specific type of involvement, whereas human disorders consist of complex biological processes and diverse metabolic pathways, which have an interactive molecular basis and are affected by some environmental factors (Bersanelli et al., 2016; Sun and Hu, 2016). Therefore, these studies are relatively simple for that kind of analysis (Chen et al., 2012). Multi-omics technologies can help to achieve a holistic view and information on these complex mechanisms by studying different layers of omics information in a multidimensional network simultaneously. Multi-omics provides an analysis system to develop knowledge of the underlying interactive molecular basis to determine the pathophysiology of disorders and their associated longitudinal effects more accurately (Figure 2; Sun and Hu, 2016). Indeed, precision medicine may benefit from integrative omics providing data along with other helpful methods such as electronic health records, imaging, and other integrative software tools (Huang et al., 2017). Multi-omics may help to access novel approaches for diverse phases of the disorder including prevention, early diagnosis, and treatment. For instance, in the field of cancer treatment, the comprehensive single-cell survey may be effective to clarify underlying different biological and molecular basis finding in regional subdivisions of it (Sun and Hu, 2016). Integrative omics data derived from the cancer genome atlas have been shown to be helpful in profiling the druggability of cancer comprehensively (Sengupta et al., 2018). In addition, multi-omics data can provide novel approaches targeting drug resistance, which can be related to personalized medicine (Bock et al., 2016). In the next part, we are going to explain multi-omics technologies targeting T2DM and HF as two major human disorders in addition to stating five different layers of it, namely, genomics, metabolomics, transcriptomics, proteomics, and epigenomics.

## Multi-Omics Studies Targeting T2DM and HF Common Mechanisms

Multi-omics technologies have promoted the knowledge of pathways of different disorders such as T2DM, obesity, cancer, and many others. Measurement of some relevant biomolecules can be helpful in the investigated environment. For instance, in metabolic disorders, important metabolites such as relevant biomolecules can reflect metabolic states (Chen et al., 2020). One of the growing fields for studying different metabolites (low molecular weight molecules) is metabolomics. Metabolomics assay techniques such as nuclear magnetic resonance (NMR) spectroscopy, MS, and chromatography help us to investigate the expression and posttranslational modification of transcription factors during different pathologic conditions (Kappel et al., 2016). Biologically, according to energy requirement, alterations in cardiac function occur. Herein, systems biology approaches such as metabolomics provide data to reveal how these metabolic changes during T2DM affect myocardial redox function (Cortassa et al., 2020). Indeed, both environmental influences and individual predisposition could be reflected by metabolite profiling, which makes it a useful method for investigating the pathophysiology of different diseases (Padberg et al., 2014). Herein, a study on multi-omics analysis on T2DM db/db mice aimed to characterize alterations in cardiac function, by investigating the effects of transferring longevity-associated-variant (LAV) of the human bactericidal/permeability-increasing fold-containing-Family-B member 4 (BPIFB4) gene. It was shown that alterations in heart lipid metabolism considering elevated levels of FA, acyl-carnitine, sphingolipid, ceramides, diacylglycerol, and triacylglycerides were notable metabolic changes in metabolic phenotyping investigations. Also, they were associated with impaired mitochondrial function with exertion of some effects on insulin sensitivity pathways (Faulkner et al., 2020). According to the results of previous studies, any disruption in lipid metabolism leads to deleterious effects on the diabetic heart. Understanding and managing this complication become possible by lipidomics profiling, which means observing lipids and providing an insight into their interactions. Indeed, lipidomics, which quantitatively analyzes lipids, is considered a subset of metabolomics (Tham et al., 2018). In recent times, a clear correlation between abnormal triglyceride accumulation and heart dysfunction, which are more prevalent in patients with obesity and diabetes, has been demonstrated. Lipidomics analysis of a diabetic heart demonstrated the important role of phospholipids in the development of this pathological condition (Dong et al., 2017). Lipid classes were also affected by the alterations in some lipid metabolites of the cellular membrane (glycerophospholipid and cardiolipin lipid classes), which suggests membrane composition changes with notable influences on cardiac functions. For instance, impaired cardiolipins have been shown to contribute to reduced contractility with effects on the mitochondrial electron- transport chain. Other associated changes were sarcomere rearrangement and loss of mitochondrial cristae and matrix volume (Paradies et al., 2019). One of the proposed mechanisms between DM and HF is attributed to excess activation of renin/angiotensin as well as sodium-glucose transporter 2, which can lead to exacerbation of overload volume in HF. Increased systemic inflammation is an additional common mechanism in DM and HF pathogenesis that results in an exacerbation of cardiac extracellular matrix remodeling and endothelial dysfunction. Decreased bioavailability of nitric oxide and microvascular dysfunction are consequences of hyperglycemia that are accompanied by production of glycated end products. Impaired function of myocyte mitochondria occurs following insulin resistance-induced increase in free FA consumption in the myocardium and toxic lipid intermediates accumulation (Hanff et al., 2021). Although several data on cellular and molecular mechanisms underlying cardiovascular disease have been obtained using genomics analysis techniques, more promising results are provided by proteomics technology. Proteomics outcomes together with genomics data broaden the knowledge of specific pathways involved in heart failure. For instance, identifying the alterations that occur in association with mitochondrial energy metabolism, stress response, and mitochondrial signaling is performed by proteomics. Indeed, cardiac proteome changes serve as an indicator of DC and are useful in assessing the consequences of different therapies for DM complications associated with heart disorders. Altogether, proteomics approaches have the potential to be applied in different study disciplines, from animal studies to cell culture systems, to address different questions (Karthik et al., 2014). Regarding multi-omics technology, utilizing LAV-BPIFB4 with stromal cell-derived factor-1/C-X-C chemokine receptor-type4 dependent effects influences cardiac contractility of diabetic db/db mice. Mitochondrial metabolism and function can be affected in this intervention with cardioprotective effects. RNA-seq analysis has also been performed to find transcriptional changes in metabolism- and immune-related genes, but alterations in protein level could not be observed in the progression of DC, which suggested the limited effects of metabolic enzyme expression at the time point of the study. Taken together, this study reveals the possible therapeutic benefits of LAV-BPIFB4 gene transfer due to its positive effects on mitochondrial FA handling along with energy production in treated mice (Faulkner et al., 2020). In the other multi-omics study, the effects of environmental glucose levels on the miRNA–mRNA dynamics have been shown by using high-throughput sequencing and qRT-PCR. The results suggest that miRNA-mediated gene regulation can be a useful biomarker for treatment and can promote knowledge of the development of diabetes (Chen et al., 2020). As mentioned before, cardiac dysfunction in the diabetic state can be related to mitochondria uncoupling along with mitochondrial-electron transport chain leakage (Varma et al., 2018). In this regard, multi-omics approaches regarding lipidomics and proteomics with functional investigations can be promising tools to understand the cause of mitochondria dysfunction and to use the underlying knowledge for personalized treatments of different disorders such as diabetes and HF (Kappler and Lehmann, 2019). On the other hand, as stated earlier, inflammatory responses have effects on CAN (Rodriguez-Saldana, 2019). In another study on the identification of key genes involved in DM, it was established that underlying immunity and inflammation pathways have important roles in DM. This analysis using the Linked Omics database has shown that MMP9 (an important hub gene) can be helpful for the treatment of DM suggests as an inflammatory regulator in diabetic peripheral neuropathy. Herein, MMPs have notable effects on immunity responses by regulating cytokine function (Jian and Yang, 2020). Multi-omics analysis of integrative data from population-based genetic analysis, miRNA expression data collection, and DNA methylation has found several cardiometabolic-related miRNAs, which have roles in lipid metabolism. These miRNAs could be also defined as potential biomarkers for the earlier diagnosis of T2D and CHD or their development pathways. However, more research studies are needed to explain the connection between elevated blood glucose and these miRNAs (Mens et al., 2020). Some of the other studies performed are shown in Table 1.

**TABLE 1:** Examples of recently performed multi-omics studies on diabetic heart disease.

	**Article title**	**Type of studied disease model**	**Omics technique**	**Result**	**References**
1	Posttranslational modulation of FoxO1 contributes to cardiac remodeling in post-ischemic heart failure	Acute myocardial infarction mice model	Metabolomics	Post translationally modification of cardiac FoxO1 by diabetes and ischemia	Kappel et al., 2016
2	Quantitative Proteomic Analysis of Diabetes Mellitus in Heart Failure With Preserved Ejection Fraction	Proteins with HFpEF and DM	Proteomics	Identifying proteins related to lipid metabolism, inflammation, and oxidative stress that are differentially expressed in patients with diabetes with HFpEF	Hanff et al., 2021
3	A proteomics approach to identify the differential protein level in cardiac muscle of diabetic rat	Diabetic rats	Proteomics	Identifying common mechanisms linked between DM and heart disease	Karthik et al., 2014
4	Investigation of the Protective Effects of Phlorizin on Diabetic Cardiomyopathy in db/db Mice by Quantitative Proteomics	db/db diabetic mice	Quantitative Proteomics	Probable protective effects of Phlorizin against diabetic cardiomyopathy	Cai et al., 2013
5	Mitochondrial dysfunction in the type 2 diabetic heart is associated with alterations in spatially distinct mitochondrial proteomes	Mitochondrial dysfunction in T2DM heart in db/db mice	Quantitative Proteomics	Association of mitochondrial dysfunction in T2DM heart with specific subcellular locale	Dabkowski et al., 2010
6	Proteomics of the Rat Myocardium during Development of Type 2 Diabetes Mellitus Reveals Progressive Alterations in Major Metabolic Pathways	Zucker diabetic fatty rat heart	MS based proteomics	Up-regulation of fatty acid degradation from onset to late T2DM	Edhager et al., 2018
7	Multi-proteomic approach to predict specific cardiovascular events in patients with diabetes and myocardial infarction: findings from the EXAMINE trial	Patients with diabetes and a recent MI	Proteomics	Better reclassification and risk prediction and event, better targeted treatment decisions and risk assessment	Ferreira et al., 2021
8	Changes of myocardial lipidomics profiling in a rat model of diabetic cardiomyopathy using UPLC/Q-TOF/MS analysis	Diabetic cardiomyopathy model in rats	UPLC/Q-TOF/MS	The suggestion of some changes in lipid biomarkers involved in hypertrophy of diabetic cardiomyopathy and cardiac dysfunction	Dong et al., 2017
9	Lipidomic Profiles of the Heart and Circulation in Response to Exercise versus Cardiac Pathology: A Resource of Potential Biomarkers and Drug Targets	Mice with physiological cardiac remodeling	Lipidomics	– Highlighting lipid profile adaptations in response to training versus pathology – Providing a resource to investigate of potential therapeutic targets and biomarkers	Tham et al., 2018
10	Diabetes changes gene expression but not DNA methylation in cardiac cells	Diabetic mice	Transcriptome analysis	Revealing differentially regulated gene programs associated with diabetes biological processes	Lother et al., 2021
11	Transcriptomic analysis of the cardiac left ventricle in a rodent model of diabetic cardiomyopathy: molecular snapshot of a severe myocardial disease	Diabetic cardiomyopathy model in rats	Transcriptomic analysis	Providing a molecular overview to processes leading to myocardial disease in diabetes	Glyn-Jones et al., 2007
12	Cardiac transcriptome profiling of diabetic Akita mice using microarray and next generation sequencing	Akita heart model in mice	Transcriptomic analysis	Providing a platform for future targeted studies to investigate genes involved in Akita heart and diabetic cardiomyopathy	Kesherwani et al., 2017
13	Divergent transcriptomic profiles in skeletal muscle of diabetics with and without heart failure	Patients with T2DM	Transcriptomic analysis	Confirming distinct transcriptome profiles of skeletal muscle in DM patients with and without HF	Wood et al., 2021

*FOXO1, forkhead box O1; HFpEF, heart failure with preserved ejection fraction; T2DM, type 2 diabetes mellitus; HF, heart failure; DM, diabetes mellitus.*

## Conclusion and Future Perspectives

Taken together, HF, an important comorbidity/complication of diabetes, has a high incidence and a high mortality rate in diabetic patients (Bertoni et al., 2004; Bowes et al., 2019). HF most commonly occurs following other CV events such as ischemia and hypertension (Bowes et al., 2019). Patients with DM and HF have specific manifestations of metabolic, structural, and neurohormonal abnormalities, which may worsen HF outcomes (Dei Cas et al., 2015). Alterations in insulin signaling, lipid accumulation, mitochondrial dysfunction, higher AGEs, and oxidative stress are some of the underlying mechanisms of this DC (Tarquini et al., 2011; Voors and van der Horst, 2011), which were explained before. Herein, lifestyle modification, blood glucose control, considering and eliminating risk factors for CV events, and treatment of HF can be helpful to achieve better outcomes regarding DC (Trachanas et al., 2014). On the other hand, omics studies have been developed as high-throughput technologies that have revolutionized medical research. Each layer of the omics studies can provide an associated list of differences with the disorder. Data derived from the single layer of omics data could be used as the biological markers of disease progression along with providing knowledge about disease pathways or processes. For instance, in the field of epigenomics, differentially methylated DNA regions can be helpful as disease indicators in the different disorders of metabolic syndrome or CV disease (Kim et al., 2010; Hasin et al., 2017). In addition to the benefits associated with omics technologies, multi-omics techniques provide improved and integrated characterization of biological pathways through different omics layers (Argelaguet et al., 2018). Drug discovery using omics technologies can be helpful through non-invasive data collection to manifest disease progression in order to achieve direct and translatable phenotype modeling of disorder (mapping disease phenotypes). It could also show the molecular basis and biomarkers of disorders. These technologies can also be effective to identify at-risk individuals, epidemiology, and tailoring cures as personalized medicine (Cisek et al., 2016). More knowledge of biological pathways utilizing multi-omics data can reveal the relationship between a disease and environmental factors. That may lead to earlier and more accurate diagnosis utilizing biomarkers of diseases along with more developed pharmacological and improved interventions for specific groups of patients (Koh and Hwang, 2019). T2DM and HF as two major disorders with some common underlying mechanisms that can also benefit from these advantages of omics/multi-omics approaches targeting their mentioned common pathways (Faulkner et al., 2020). Indeed, the development of next-generation sequencing along with mass-spectrometric techniques strategies provide large-scale research on whole cellular systems (Chakraborty et al., 2018). Nevertheless, efficiently utilizing multi-omics data requires proper data combination of different omics layers as well as effective bioinformatics strategies and standardized protocol (Koh and Hwang, 2019). A large amount of data and lack of related research for prioritizing tools and analysis utilized in multi-omics approaches, as well as lack of established standards for data filtering, are other challenges associated with multi-omics techniques (Subramanian et al., 2020).

## Author Contributions

All authors listed have made a substantial, direct and intellectual contribution to the work, and approved it for publication.

## Conflict of Interest

The authors declare that the research was conducted in the absence of any commercial or financial relationships that could be construed as a potential conflict of interest.

## Publisher’s Note

All claims expressed in this article are solely those of the authors and do not necessarily represent those of their affiliated organizations, or those of the publisher, the editors and the reviewers. Any product that may be evaluated in this article, or claim that may be made by its manufacturer, is not guaranteed or endorsed by the publisher.

## Abbreviations

ACE: angiotensin converting enzyme
AGEs: advanced glycation end products
AIF: apoptosis-inducing factor
ATP: adenosine triphosphate
BPIFB4: bactericidal/permeability-increasing fold-containing family B member 4 (BPIFB4)
CAN: cardiac autonomic neuropathy
CK: creatine kinase
CoQ: coenzyme Q
CV: cardiovascular
DM: diabetes mellitus
ER: endoplasmic reticulum
FA: fatty acids
GWASs: genome-wide association studies
HbA1c: hemoglobin A1c
HF: heart failure
IGF-1: insulin-like growth factor 1
IL: interleukin
LAV: longevity-associated variant
MiRNAs: microRNAs
MS: mass spectrometry
NMR: nuclear magnetic resonance
NADPH: nicotinamide adenine dinucleotide phosphate
NF- κ B: nuclear factor kappa-light-chain-enhancer of activated B cells
NO: nitric oxide
NOX: nicotinamide adenine dinucleotide phosphate- oxidases
*O*-GlcNAc: O-linked*N*-acetylglucosamine
PARP-1: poly [ADP-ribose] polymerase 1
PI3K: phosphoinositide 3-kinase
PKA: protein kinase a
PKC: protein kinase c
PP1: protein phosphatase 1
RAAS: rennin-angiotensin-aldosterone system
RNS: reactive nitrogen species
ROS: reactive oxygen species
SR: sacoplasmic reticulum
T2DM: type 2 DM
TNF: tumor necrosis factor.
